# From pedigrees to practicality: an interactive tool for cat breeding

**DOI:** 10.3389/fvets.2026.1749164

**Published:** 2026-02-20

**Authors:** Steven R. Talbot, Nora Weegh, Miriam Heider, André Bleich

**Affiliations:** Hannover Medical School, Institute for Laboratory Animal Science, Hannover, Germany

**Keywords:** feline breeding, inbreeding monitoring, pedigree management, shiny web application, virtual breeding simulation

## Abstract

Breeding programs for companion animals are increasingly relying on accurate inbreeding coefficients to maintain genetic diversity and limit the occurrence of deleterious traits. To bridge the gap between advanced genetic tools and everyday breeder practice, we developed pawlineR, a streamlined, browser-based application that leverages R libraries to automate pedigree entry, validate parent-offspring relationships, and calculate the inbreeding coefficient (*F*) in real time. A historical dataset of 70 cats was used to evaluate the tool (pawlineR). Among these animals, 24 showed moderate to high inbreeding values (0.063–0.375). “Virtual breedings” allow users to simulate hypothetical offspring and test potential inbreeding risks before committing to real matings, promoting proactive decision-making. Manual validation using Wright’s formula confirmed the accuracy of the tool’s calculations. Accuracy depends on pedigree completeness. By offering user-friendly interfaces and stable, long-term pedigree storage, this application helps cat breeders track *F* across generations. Overall, the tool reduces technical barriers, supports routine breeding management, and can be extended to other species.

## Introduction

Cat breeding programs often prioritize aesthetic standards and specific traits, sometimes at the expense of genetic diversity. To safeguard against excessive accumulation of deleterious alleles, breeders track the inbreeding coefficient (*F*), which estimates the probability that two alleles at a locus are identical by descent (IBD) (1, 2). High inbreeding levels can compromise fitness, reducing fertility or increasing susceptibility to hereditary diseases. This risk underscores the importance of structured pedigree management in maintaining the genetic variability necessary for robust feline populations (3).

Despite growing awareness, many breeders struggle to implement efficient inbreeding-monitoring strategies. Although advanced R libraries [e.g., ribd (4), pedtools (5)] provide reliable path-tracing algorithms for *F* calculation (6, 7), breeders may find script-based interfaces challenging. Inconsistent systems for data storage and manual entry can also create inconsistencies, leading to overlooked relationships or incomplete genealogical records.

To address these challenges, we designed a web-based platform that merges specialized R functions into a streamlined dashboard, called pawlineR. Our application accommodates secure data entry, checks parental consistency (e.g., preventing an animal from being listed as both father and mother), and instantly calculates *F* for each newly registered or virtual offspring. The primary target users are cat breeders, both hobbyists and professional catteries, as well as veterinarians advising on breeding decisions, researchers studying feline genetics, and institutional facilities maintaining cat colonies. This solution aims not only to reduce inadvertent increases in inbreeding, which is an issue particularly acute in smaller breeding circles, but also to promote transparency and comparability across multiple catteries. Ultimately, by enabling breeders to make data-driven decisions about mating pairs, the system contributes to healthier cat populations and more sustainable breeding practices over the long term.

## Methods

### Implementation and data structure

The application is built as a Shiny app^1^ in R (v4.4.1), coupled with a lightweight SQLite database that stores each animal’s unique identifier, sex, inbreeding value, and pedigree links. Newly registered individuals are verified against existing records to avoid duplicates, and unknown parents are assigned “founder” status. Such controls maintain data quality and enable the continuous expansion of the pedigree while preventing logical conflicts (e.g., identical animals serving as sire and dam). All pedigree data are entered by users either manually or via spreadsheet import. The application performs structural validation (e.g., preventing sex mismatches or circular parentage) but cannot verify biological parentage. Users are encouraged to cross-reference entries with official pedigree certificates or, where available, DNA-based parentage verification. The minimum information required for entering an animal is a unique identifier and sex (male, female, or unknown). Parent information is optional but may limit the calculation of inbreeding coefficients.

### Inbreeding coefficient calculation

Whenever a new animal is added or updated, the application reconstructs its ancestral paths and applies Wright’s formula: (1, 7).


$$F(A)=\sum_{\mathrm{all}\;\text{common ancestors}\;X}{\left(\frac{1}{2}\right)}^{\left(n_{A}+n_{B}+1\right)}$$ (1)

Where *n_A_* and *n_B_* denote the number of parent-offspring steps from X to A through each parental line. This method is implemented behind the scenes via relatedness-focused functions, enabling breeders to compute *F* without manual coding. The pedigree object is built dynamically using the R package pedtools and calculates each planned kitten’s inbreeding coefficient using the inbreeding function. The resulting *F*-values are stored in the SQLite relational database. The ribd package is also implemented to calculate kinship metrics. Parent–child edges are passed through igraph and plotted in the application’s UI, where vertex shape/color encodes sex and the calculated *F* values. The inbreeding coefficient is calculated using all available pedigree information, with no (minimum or maximum) artificial limit on generation depth. The accuracy, therefore, depends on pedigree completeness. This simplified formula assumes that founder individuals have an inbreeding coefficient of zero (FA = 0). The ribd package can accommodate inbred founders when their *F*-values are known.

Inbreeding coefficients were classified according to thresholds corresponding to specific pedigree relationships (1): *F* < 0.0625 (low; below first-cousin mating) (6), *F* = 0.0625–0.125 (moderate; first-cousin to half-sibling equivalent), *F* = 0.125–0.25 (high; half-sibling to full-sibling equivalent), and *F* ≥ 0.25 (very high; full-sibling or parent-offspring equivalent). These thresholds align with empirical observations that inbreeding depression becomes biologically significant above *F* = 0.10 (8), with severe fitness consequences documented at *F* ≥ 0.125 in mammalian populations (9).

### Application workflow

Breeders can inspect, edit, or revise animal records. For new matings, they select a sire and dam from the database. The software then checks for inconsistencies and calculates *F* for the prospective offspring. “Virtual breeding” features allow hypothetical offspring to remain uncommitted until relevant details (e.g., sex) are confirmed. Users can export their pedigree data into a spreadsheet format for external storage and analysis. These files can also be reimported into the application. The pawlineR app is freely available for non-commercial use on GitHub and can be used from within R/Rstudio/posit^2^ with the appropriate packages. For a more permanent solution, users can run the app on a private Shiny server, e.g., on a Virtual Private Server (VPS). The following results were calculated with an example set of cat breeding data, which are also provided in the repository. The application displays a color-coded risk interpretation (None/Low/Moderate/High/Very High) for each computed *F*-value, aiding breeders in quickly assessing potential inbreeding concerns.

## Results

### General statistics

A historical dataset of 70 cats was used to test the system’s capacity for storing and computing *F*. Of these, 26 were male, 34 were female, and 10 had an unknown sex due to incomplete documentation. Inbreeding coefficients above zero were found in 24 animals, ranging from 0.0625 to 0.375, indicating moderate to high inbreeding in specific lines. Based on established genetic relationships, *F* < 0.0625 indicates low inbreeding, *F* = 0.0625–0.125 moderate inbreeding (first-cousin to half-sibling equivalent), *F* = 0.125–0.25 high inbreeding, and *F* > 0.25 very high inbreeding.

#### Virtual breeding

The application’s “virtual breeding” feature enables users to simulate pairings among established animals, creating hypothetical offspring for planning or research purposes. While such virtual animals can remain purely hypothetical, breeders can commit them to the permanent database at their discretion, for instance, after the actual mating occurs or when offspring details are confirmed. This function allows breeders to preview the expected inbreeding coefficient of potential offspring before committing to actual matings, thus aiding strategic decisions about future breedings.

**Figure 1:**
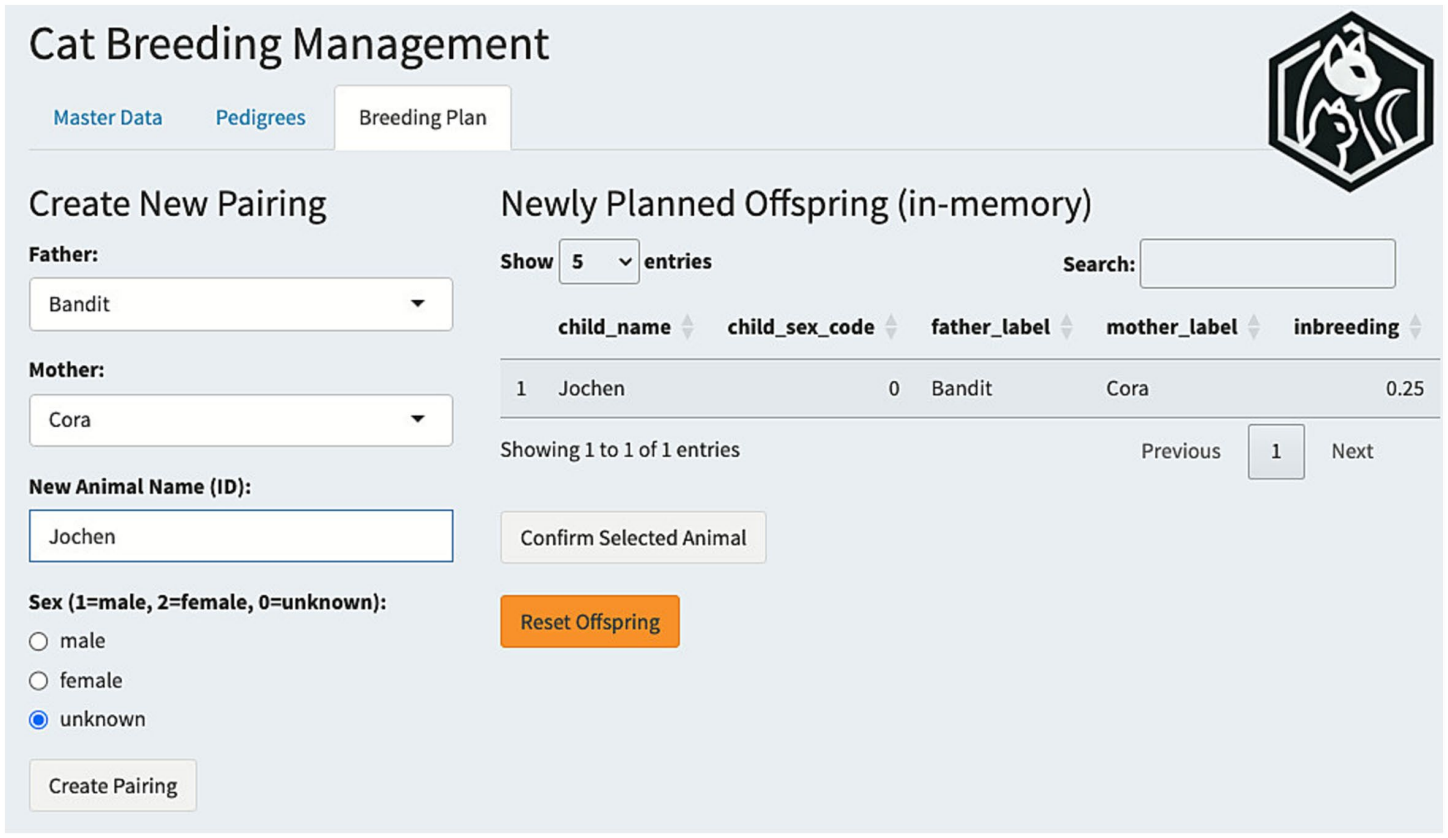
Breeding plan panel for virtual breeding. Users input sire and dam, generate a proposed offspring, and see *F* in real time. Virtual animals can remain hypothetical or be permanently added for monitoring across multiple generations. From the panel, individual pedigrees can be visually inspected. Reproduced with permission.

In one illustrative example, the animals “Bandit” (male) and “Cora” (female) from the example data were paired within the application to produce a new virtual offspring, “Jochen.” Because Cora is Bandit’s mother, the resulting offspring has a theoretical inbreeding coefficient of 0.25 (25%). This value was calculated in real-time by the tool, demonstrating how mother-son pairings substantially elevate *F*-values (Figure 1). From here, further virtual breedings in this animal line showed a steady increase in *F* (Table 1).

**Table 1:** Example of “virtual breeding” in pawlineR.

Virtual offspring	Sex	Father	Mother	*F*
Jochen	0	Bandit	Cora	0.25
Merle	0	Jochen	Cora	0.375
Carl	0	Bandit	Merle	0.3125
Luise	0	Carl	Merle	0.5

### Manual validation of the inbreeding coefficient

To validate the computation of the inbreeding coefficient (*F*) according to Wright’s formula Equation 1, *F* was validated manually for two cases.

#### High relatedness

**Figure 2:**
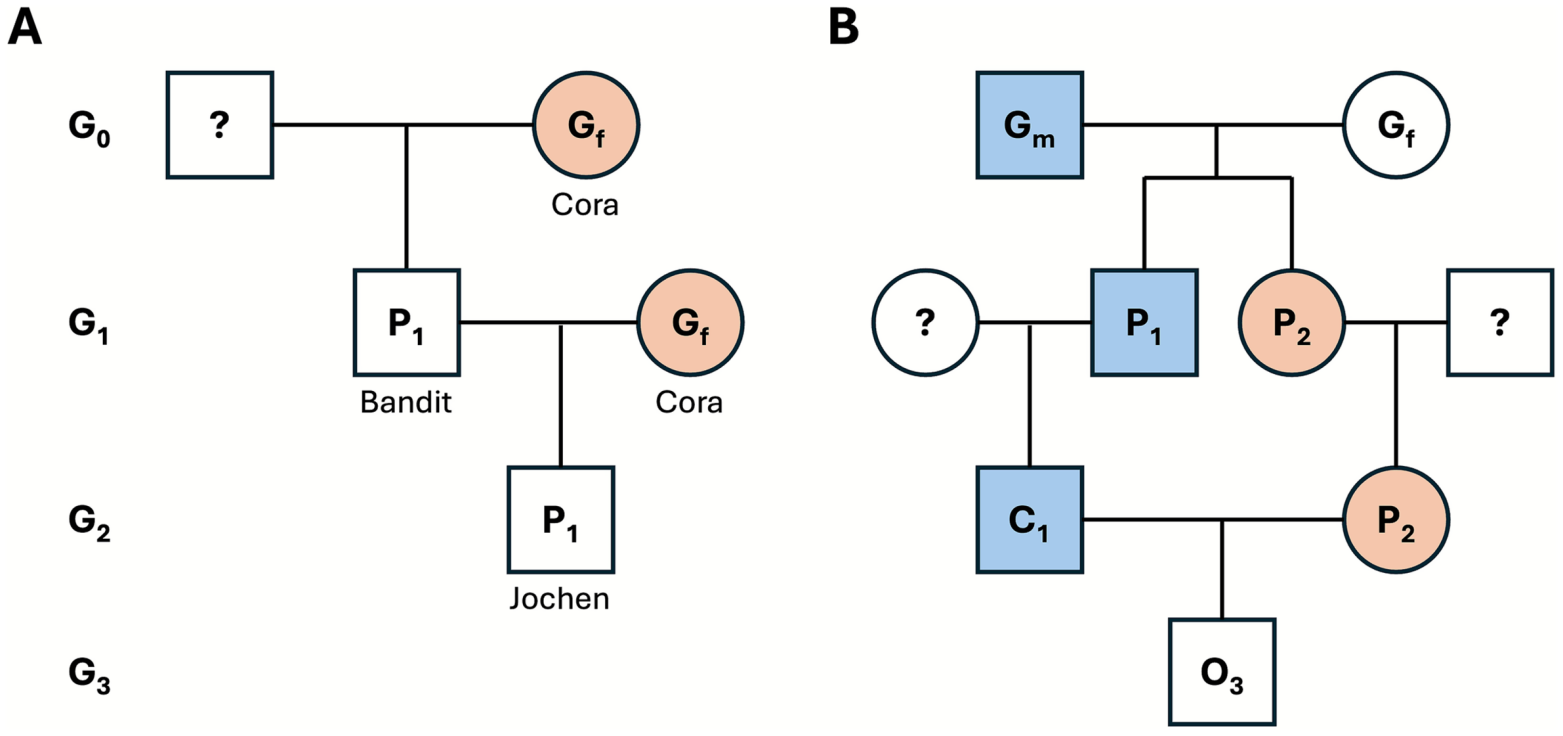
Pedigree scenarios are used for illustrative inbreeding calculations. **(A)** Mother-son mating (dam *Cora* × son *Bandit*) yielding an inbreeding coefficient of *F* = 0.25. **(B)** First-cousin mating (*C_1_* × *P_2_*), yielding *F* = 0.0625. Squares and circles represent males and females, respectively. Duplicated female ancestors are shaded orange. Relevant (but not identical) ancestors of the male line are shaded blue to highlight the common ancestral paths that generate inbreeding. Generational tiers (G_0_–G_3_) are indicated on the left, while “?” denotes an unknown founder.

Simulation of a mother-son matching (Figure 2A). The dam (Cora**♀**) was a founder (*F*_c_ = 0), and her son (Bandit) back-mated to her. The only common ancestor of the virtual offspring (Jochen) was Cora herself, with path lengths n_1_ = 1 (Jochen ➔ Cora) and n_2_ = 0 (Cora ➔Cora). Thus,


$$F={\left(\frac{1}{2}\right)}^{\left(1+0+1\right)}[1+0]={\left(\frac{1}{2}\right)}^{2}=\frac{1}{4}$$

Consequently, the inbreeding coefficient *F* for the offspring of this mother-son pairing was 0.25 (25%), which matched the virtual breeding result from before.

#### Moderate relatedness

Two unrelated founders (grandsire G_m_ and granddam G_f_) produced a son (P₁) and a daughter (P₂). Their respective progeny (C₁ and C₂) were first cousins and were mated, giving offspring O₃. Only two common ancestors existed (G_m_ and G_f_), each a founder with FA = 0 (Figure 2B). For both ancestors the parental paths were n_1_ = 2 (C₁ ➔ A) and n_2_ = 2 (C₂ ➔ A), resulting in


$$F=2{\left(\frac{1}{2}\right)}^{\left(2+2+1\right)}=2{\left(\frac{1}{2}\right)}^{5}=\frac{1}{16}=0.0625$$

This example can also be reproduced in the virtual breeding section of pawlineR.

**Figure 3:**
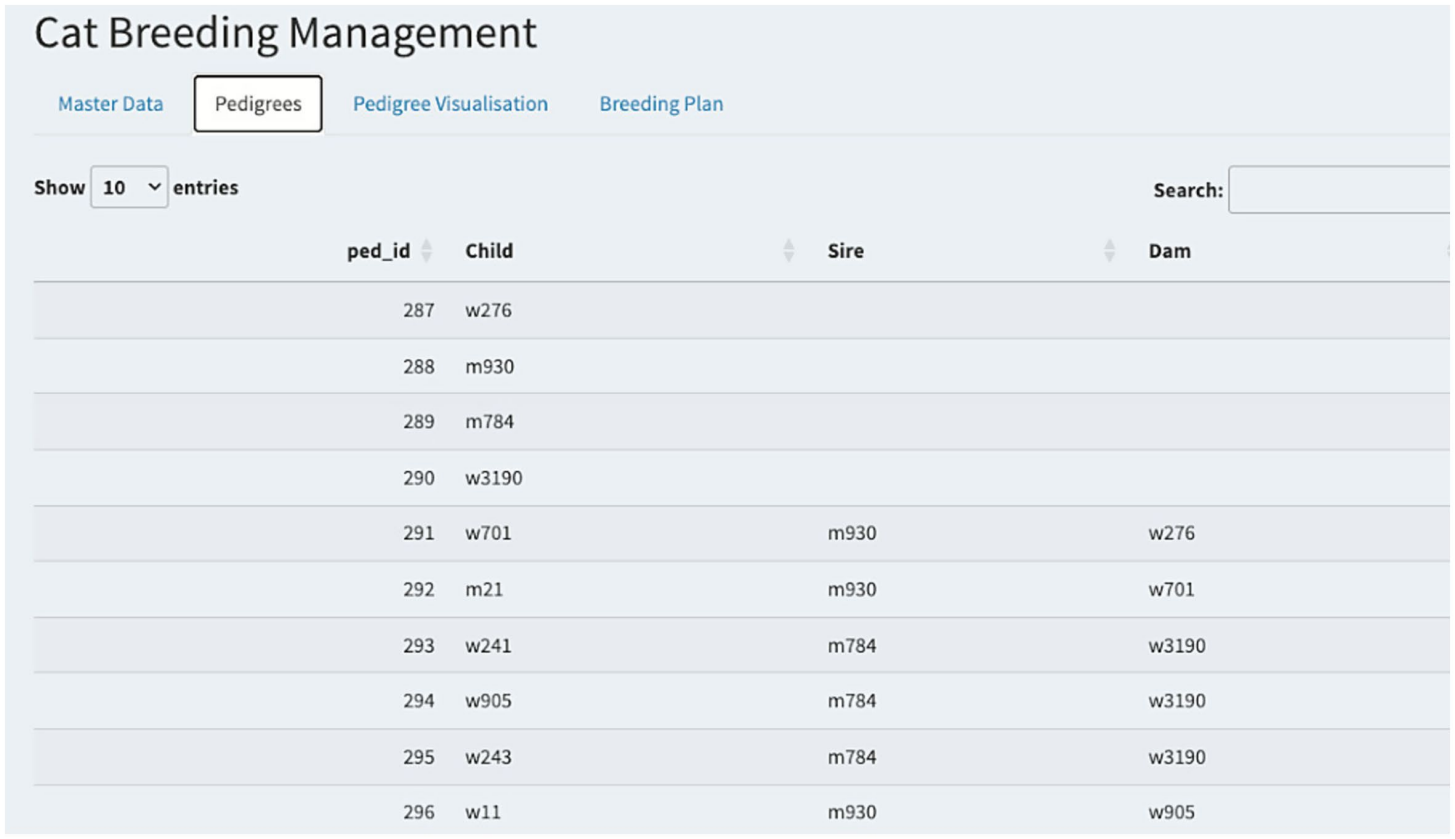
Example of pedigree data visualization within the application interface. For each ID, available pedigree details (specifically sire and dam IDs) are displayed. When sufficient data are provided, users can interactively explore the pedigree structure directly from the main panel. Reproduced with permission.

These straightforward examples illustrate how Wright’s formula can be applied once the appropriate common ancestor(s) and generation counts are identified. Users have the flexibility to incorporate their own data into the application. Sample datasets used in this study are available within the corresponding GitHub repository, providing reference material for new users. A template table is provided to streamline the process of uploading new records. Additionally, Figure 3 illustrates how pedigree data are visually represented within the application interface. The application can easily be expanded to other species and users.

## Discussion

By merging established pedigree libraries (pedtools and ribd) into a single application, we have simplified data entry, pedigree verification, and *F* computation. Non-technical users can rapidly identify risky matings and maintain detailed breeding records, facilitating more effective long-term management of genetic diversity (10). The integration of “virtual breeding” further supports proactive strategies, enabling breeders to prevent high *F* levels by modeling hypothetical pairings in advance.

Nevertheless, results depend on complete and accurate lineage records. Any missing or erroneous genealogical data may underestimate the inbreeding coefficient (*F*) (11). Additionally, while pedigree-based *F* offers valuable insights, genomic marker data can yield finer-grained analyses of homozygosity (12). As an initial step, however, this tool meets many catteries’ practical needs, helping breeders balance daily operational demands with the broader goal of sustained cat population health. A further limitation concerns technical accessibility: while the graphical interface eliminates the need for R programming during daily use, the initial setup requires installing R/RStudio and associated packages, which may be challenging for users without data-analysis experience. Deploying the application on a private Shiny server can mitigate this barrier by providing browser-based access without local installation.

However, beyond the advantages already described, three additional issues deserve attention. While Wright’s path-coefficient of inbreeding (*F*) is intuitive and fast, other complementary indicators, e.g., mean kinship (13) or projected effective population size, can contribute to a more complete interpretation. Mean kinship represents the average relatedness of an individual to all others in the population, and guides mate selection to preserve diversity. Effective population size (N_e_) estimates the genetic “size” of a population and indicates the rate of diversity loss (14, 15). These alternative approaches help breeders balance genetic diversity across the whole colony rather than animal by animal. In future versions, these indices could be included with a classical tabular method or by simple gene-dropping simulations. In later stages, additional data, such as phenotype-related animal health, welfare, or trait records, can be included. As these methods extend beyond practical, day-to-day breeding work, they were not included in this initial version.

From the 3R’s perspective, “virtual breeding” allows users to explore multiple pairings before any actual mating occurs. This feature effectively reduces the number of actual litters required to achieve a target genetic profile. It is also a refinement of the selection process, as it flags high-risk crosses early. Although cat breeding is not a traditional form of laboratory experimentation, the same principle of minimizing unnecessary animal production applies to companion animal welfare. It aligns the tool with contemporary ethical guidelines.

Finally, the application is a valuable asset in research planning, security, and documentation integrity. Each transaction (e.g., animal addition, deletion, or pedigree edit) is time-stamped in the relational database, providing a data trail that can be exported as plain CSV for archiving alongside publications or breeding reports. Routine database back-ups (or downloadable snapshots) ensure that lineage data used to justify mating decisions remains verifiable. This backup procedure safeguards both scientific reproducibility and the authenticity of pedigree.

Taken together, pawlineR shows that robust population-genetic analytics, transparent record-keeping, and day-to-day breeding logistics can be integrated without sacrificing usability. By embedding pedigree algorithms behind an intuitive interface, the application turns complex calculations into actionable flags and recommendations, while its database preserves decisions for later audit or replication.

Because the code base is modular and open to community extensions, the platform can evolve in steps with emerging best practices. We therefore encourage breeders, veterinarians, and researchers to adopt and refine this framework, so that companion-animal programs can move toward a shared, reproducible standard of genetic stewardship and, ultimately, healthier, more diverse cat populations.

## Data Availability

The pawlineR Shiny application and source code are released under the Free-Use-Non-Commercial-Share-Alike Licence (FUNCSA) v1.0. The pedigree datasets and example data are licensed under Creative Commons Attribution-NonCommercial-ShareAlike 4.0 International (CC BY-NC-SA 4.0). Users may copy, adapt, and share the data for non-commercial purposes only, with attribution. Any commercial use of the software or data requires the Licensor’s prior written permission. GitHub repository: https://github.com/mytalbot/pawlineR.

## Footnotes

1
https://www.shinyapps.io

2
https://github.com/mytalbot/pawlineR
